# Four Novel HLA‐B and HLA‐C Alleles Identified in Brazilian Individuals

**DOI:** 10.1111/tan.70681

**Published:** 2026-03-24

**Authors:** Raquel Aparecida Fabreti‐Oliveira, Cíntia Keilla Fabreti de Oliveira, Evaldo Nascimento

**Affiliations:** ^1^ Department of Molecular Biology IMUNOLAB, Laboratory of Histocompatibility Belo Horizonte Minas Gerais Brazil; ^2^ Postgraduate Program in Health Sciences Faculty of Medical Sciences of Minas Gerais Belo Horizonte Minas Gerais Brazil

**Keywords:** Brazilian population, new allele, NGS, REDOME

## Abstract

Identification of the four novel HLA alleles in Brazilian individuals.

HLA alleles arise through diverse genetic mechanisms, reflecting the high polymorphism of the HLA loci [1]. According to the IPD‐IMGT/HLA Database version 3.63.1 (February 2026), a total of 43,758 HLA alleles have been described, of which 29,854 belong to HLA class I. Among HLA class I alleles, 10,876 correspond to the HLA‐B locus and 9031 to the HLA‐C locus [2]. Several HLA alleles not listed as common or intermediate in the Common, Intermediate and Well‐Documented HLA Alleles Catalogue (CIWD) version 3.0.0 have nevertheless been found to be prevalent in the Brazilian population, underscoring the unique genetic composition of this admixed population [3].

The implementation of high‐resolution DNA sequencing in routine HLA typing continues to reveal previously undescribed variants, such as the four novel Class I alleles reported in this study: HLA‐B*44:02:91, HLA‐B*52:01:59, HLA‐B*57:199 and HLA‐C*07:02:164, identified in individuals from the Brazilian Bone Marrow Donor Registry (REDOME). While the variants HLA‐B*44:02:91, HLA‐B*52:01:59, and HLA‐C*07:02:164 do not alter the amino acid sequence relative to their most closely related alleles, they function as informative genetic markers for haplotype identification. In contrast, the identification of HLA‐B*57:199 represents a structural change at the second‐field level. Because the B*57 group has been extensively studied due to its association with spontaneous viral control and Abacavir hypersensitivity, any newly identified variant requires careful evaluation of the resulting epitope, given its potential impact on clinical practice [4]. The detection of nonsynonymous variants like B*57:199 helps prevent mismatches that may increase the risk of acute graft‐versus‐host disease (aGvHD) in haematopoietic stem cell transplantation (HSCT), while detailed resolution of the HLA‐C locus allows for the optimisation of KIR interactions (C1/C2) to enhance the graft‐versus‐leukaemia (GvL) effect [5]. In HSCT and solid organ transplantation, high‐resolution typing is essential for the definition of donor‐specific anti‐HLA antibodies (DSA).

Genomic DNA was extracted from peripheral blood samples. HLA typing was performed using the AllType NGS 11‐loci kit (One Lambda Inc., Canoga Park, CA, USA) on both the Ion Torrent S5 (Thermo Fisher Scientific, Waltham, MA, USA) and DNBSEQ‐G99 (MGI, Shenzhen, China) platforms. Data processing was conducted using TypeStream Visual software version 3.1.0 (One Lambda). To validate the novel alleles, sequence‐based typing (SBT) was performed using the SeCore HLA kit (One Lambda) on an ABI 3730 Genetic Analyzer (Applied Biosystems). The resulting sequences were analysed using uType software version 7.3 (Thermo Fisher Scientific). Additionally, the HLA‐B*57:199 allele was confirmed by Nanopore sequencing using the SQK‐RBK114.24 kit on a MinION Mk1D sequencer (Oxford Nanopore Technologies, Oxford, UK), with data analysed in TypeStream Visual version 3.2.0. Written informed consent was obtained from all four donors in accordance with institutional and national ethical guidelines. Characteristics of the novel HLA‐B and HLA‐C alleles were shown in Table 1.

**TABLE 1:** Characteristics of novel HLA‐B and ‐C alleles identified in four Brazilian individuals.

Novel HLA allele	Ethnic group	HLA‐	GenBank accession number	Most similar HLA allele	Nucleotide position change	Codon changed	Codon change	Amino acid residue change
B*44:02:91	Hispanic or Latin American—Brazil, South America	A*03:01:01, 32:01:01 B*07:02:01, 44:02:91 C*03:03:01, 07:02:01 DRB1*07:YDEC, 15:KZCC DRB4*01:03:01:02N DRB5*01:EZRWB DQA1*01:02:01, 02:01:01 DQB1*03:03:02, 06:EZRVX DPA1*01:03:01, 01:03:01 DPB1*04:EKAUV, 04:EDJXU	PV704036	B*44:02:01	24	17.3	ACC to ACA	Threonine
B*52:01:59	Unknown	A*32:01:01, 68:01:01 B*08:01:01, 52:01:59 C*07:01:01, 16:01:01 DRB1*03:01, 03:02 DRB3*01:BZFC, 01:BZFC DQA1*04:01:01, 05:01:01 DQB1*02:EDWXA, 04:EZSWT DPA1*01:03:01, 02:01:08 DPB1*01:EZSXC, 02:EBTJE	PV261954	B*52:01:02	126	18.3	GGG to GGA	Glycine
B*57:199	Hispanic or Latin American—Brazil, South America	A*01:01:01, 01:01:01 B*38:01:01, 57:199 C*07:01:01, 12:03:01 DRB1*03:ZVPY, 13:01 DRB3*01:FGVMZ, 02:FBJCW DQA1*01:10:01, 05:01:01 DQB1*02:EDWXA, 06:FGVMV DPA1*01:03:01, 02:01:01 DPB1*14:FFZMJ, 15:ESDFP	PX068457	B*57:01:01	1081	337	ACA to GCA	Threonine to Alanine
C*07:02:164	Hispanic or Latin American—Brazil, South America	A*03:01:01, 68:01:02 B*07:02:01, 40:04:01 C*03:04:01, 07:02:164 DRB1*04:05:01, 04:11:01 DRB4*01:EUHVF, 01:EUHVF DQA1*03:01:01, 03:03:01 DQB1*03:EDZDA, 03:EDZDA DPA1*01:03:01, 02:01:01 DPB1*04:EKAUX, 11:01:01	PQ817130	C*07:02:01	1080	336.3	TCT>TCC	Serine

The names HLA‐C*07:02:164, HLA‐B*52:01:59, HLA‐B*44:02:91, and HLA‐B*57:199 have been officially assigned by the WHO Nomenclature Committee for Factors of the HLA System in March 2025, April 2025, June 2025, and September 2025, respectively. This follows the agreed policy that, subject to the conditions stated in the most recent Nomenclature Report, names will be assigned to new sequences as they are identified. Lists of such new names will be published in the following WHO Nomenclature Report [6].

## Author Contributions

**Raquel Aparecida Fabreti‐Oliveira:** analysing data, sequence submission and reviewing of the manuscript. **Cíntia Keilla Fabreti de Oliveira:** analysing data and reviewing of the manuscript. **Evaldo Nascimento:** writing, reviewing and discussion of the manuscript. All authors have read and approved the final manuscript.

## Funding

The authors have nothing to report.

## Conflicts of Interest

The authors declare no conflicts of interest.

## Data Availability

The data that support the findings of this study are openly available in the IPD‐IMGT/HLA Database at https://www.ebi.ac.uk/ipd/imgt/hla/alleles/.
